# Exploring the Perceptions of Partners of Persons with Dementia on Value-Congruent Care

**DOI:** 10.1093/geroni/igaf122.1665

**Published:** 2025-12-31

**Authors:** Teuta Kadiu

**Affiliations:** University of California, Davis, Davis, California, United States

## Abstract

The purpose of this study was to explore caregivers of PwD perceptions of receiving value congruent care in encounters with healthcare professionals throughout the progression of dementia. The method was an interpretative descriptive study that included 25-partners of persons with dementia. Participants were partners caring for persons with dementia during different stages of the disease. Interviews were conducted in-person and online. Interview questions were designed based on the psychosocial caregiver integrity model, the development of which has been reported elsewhere. Several screening tools were also used to explore the patient with dementia and the partners’ health concerns as well as any relationship concerns. Interview questions explored caregivers’ perceptions of the healthcare provider’s ability to address the various concerns. Data was transcribed using AliceAI. Transcripts were interpreted using the role theory framework. From this, the results were descriptions of the elements of value congruent care, as they emerged from interviews with partners of persons with dementia. Three component themes were identified: 1) acknowledging relational concerns, 2) assessing what mattered most to the dyad, and 3) creating meaning for both. Lack of value congruent care had implications for caregivers’ socialization into the caregiving role, as well as utilization of healthcare resources. Awareness of these factors can help providers be more effective when helping socialize partners of patients with dementia into the role of a long-term caregiver.

